# Clinical Features of Neurodevelopmental Outcomes in Children with Preterm Severe Fetal Growth Restriction: A Retrospective Observational Study

**DOI:** 10.31662/jmaj.2022-0047

**Published:** 2022-06-17

**Authors:** Takahiro Motoki, Yoshitsugu Chigusa, Seiichi Tomotaki, Yosuke Kawamura, Mana Taki, Ken Yamaguchi, Masaki Mandai, Haruta Mogami

**Affiliations:** 1 Department of Gynecology and Obstetrics, Kyoto University, Kyoto, Japan; 2 Department of Pediatrics, Kyoto University, Kyoto, Japan

**Keywords:** developmental quotient, fetal growth restriction, neurodevelopment, preterm birth, small for gestational age

## Abstract

**Introduction:**

Fetal growth restriction (FGR) is a clinical condition wherein a fetus fails to achieve the expected growth potential. Although FGR is the leading cause of perinatal morbidity and mortality, there is a lack of knowledge about the long-term developmental outcomes of children who had FGR in Japan. Here, we sought to clarify the features of neurodevelopmental outcomes in preterm-born children with severe FGR (sFGR) and identify associated clinical factors.

**Methods:**

The clinical data of 26 preterm sFGR cases and 26 preterm appropriate for gestational age (AGA) cases with a similar gestational age distribution were reviewed retrospectively. Developmental quotient (DQ) scores assessed during the 1- and 2-year corrected ages using the Kyoto Scale of Psychological Development were analyzed.

**Results:**

sFGR was diagnosed at 26 (18-34) weeks of gestation, and the gestational age at delivery was 31 (25-36) weeks. The overall DQ scores of children in the sFGR group were significantly lower than those in the AGA group (80 vs. 90.5, *P* = 0.0127). Of the three areas that comprise the DQ (Postural-Motor, Cognitive-Adaptive, and Language-Social), the sFGR group only showed significantly lower DQ scores (72.5 vs. 88, *P* = 0.0255) in the Language-Social area. Both fetal body weight and fetal body weight Z score at birth significantly correlated with the DQ scores (r = 0.4912, *P* = 0.0108, and r = 0.5621, *P* = 0.0028), whereas neither the duration of fetal growth arrest nor the gestational age at birth correlated with the DQ scores (r = 0.3598, *P* = 0.0842, and r = 0.3522, *P* = 0.0776).

**Conclusions:**

Our results indicate that preterm-born children with sFGR have greater neurodevelopmental impairment than preterm-born children without FGR, specifically in terms of the DQ scores for the Language-Social area. It is imperative to encourage continuous long-term follow-up and appropriate interventions after birth.

## Introduction

Fetal growth restriction (FGR) is a clinical condition wherein a fetus fails to achieve the expected growth potential because of several factors, such as placental dysfunction, infections, or chromosomal abnormalities ^(1), (2), (3)^. Generally, FGR is diagnosed when the estimated fetal weight by ultrasound measurement is below the 10^th^ percentile ^(4)^. On the other hand, small for gestational age (SGA) refers to an infant with a birth weight below the 10^th^ percentile based on the population standard ^(4)^. Although the two are not necessarily synonymous, there is a significant overlap.

FGR is one of the leading causes of perinatal morbidity and mortality ^(1)^. In severe cases, intrauterine chronic hypoxia can cause not only fetal acidosis but also fetal death ^(1)^. Nevertheless, there is currently no proven effective treatment for FGR. The only practiced management strategies are the close monitoring of fetal well-being and the termination of pregnancy at the most appropriate time to avoid fetal acidosis and improve neonatal short-term prognosis ^(2), (5)^. Importantly, infants who had FGR and were SGA have higher rates of neurodevelopmental delay and poor cognitive outcomes ^(6), (7)^. Hence, the optimal timing for the termination of pregnancy for severe FGR (sFGR) cases should be determined not only in terms of short-term fetal prognosis but also in terms of long-term neurodevelopmental outcomes.

However, there is a lack of knowledge about the characteristics detected in the neurodevelopmental delay of children who had FGR and were SGA. Therefore, this study aimed to clarify the features of neurodevelopmental outcomes in preterm-born children with sFGR and identify associated clinical factors.

## Materials and Methods

### Definition of FGR and SGA

In this study, we defined FGR, sFGR, and SGA as follows:

- FGR: if the estimated body weight in utero by ultrasonic measurement based on the birth size standards by gestational age for Japanese neonates is below the 10^th^ percentile (−1.28 SD).
- sFGR: if the estimated body weight in utero by ultrasonic measurement is below the 3^rd^ percentile (−1.88 SD).
- SGA: if the body weight at birth is below the 10^th^ percentile.

### Ultrasonic measurement of fetal body weight in utero

The estimated fetal body weight was calculated using the following formula ^(8)^.

EFW (g) = 1.07 × BPD (cm)^3^ + 0.30 × AC (cm)^2^ × FL (cm)

EFW: estimated fetal weight

BPD: biparietal diameter

AC: abdominal circumference

FL: femur length

### Study design and patients

This retrospective observational study was approved by the Ethics Committee of Kyoto University (R3305). Electronic medical charts were used to identify and enroll patients diagnosed with FGR and babies delivered at Kyoto University Hospital between January 2011 and December 2015. The inclusion criteria were (1) patients diagnosed with sFGR and babies delivered at less than 37 weeks of gestation, and who were in fact SGA at birth, and (2) patients whose developmental quotient (DQ) was assessed between 1- and 2- years of age, corrected for prematurity. The exclusion criteria were chromosomal abnormality, multiple gestation, and congenital fetal anomaly.

**Figure 1.**
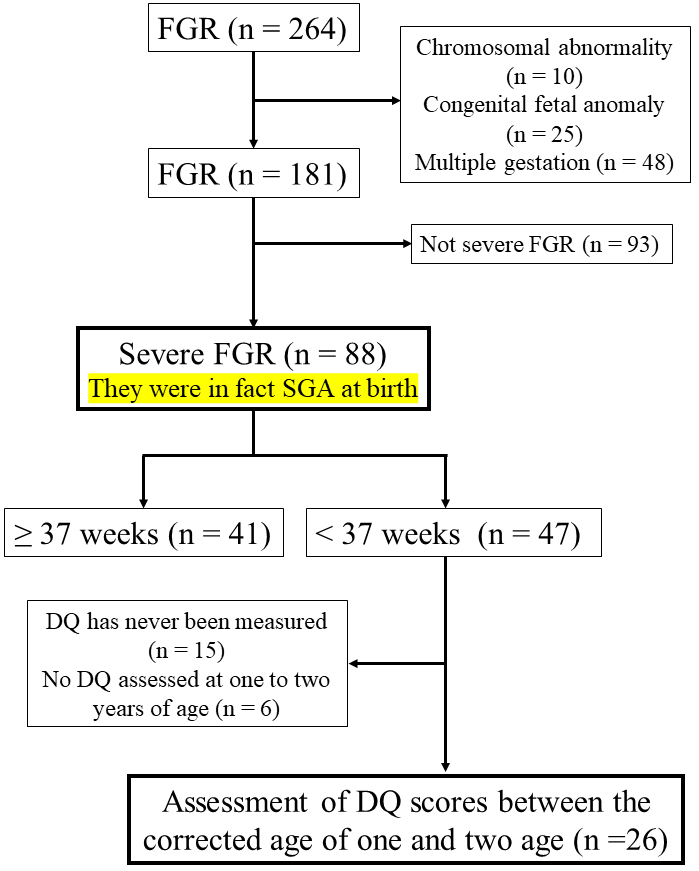
Flow diagram of patients included in the study. Fetal growth restriction (FGR) was defined as follows: the estimated body weight in utero by ultrasonic measurement and the actual body weight at birth based on the birth size standards by gestational age for Japanese neonates are below the 10^th^ percentile (−1.28 SD). Severe FGR was defined as follows: the estimated body weight in utero by ultrasonic measurement and the actual body weight at birth are both below the 3^rd^ percentile (−1.88 SD). Small for gestational age (SGA) was defined as follows: the body weight at birth is below the 10^th^ percentile. DQ: developmental quotient.

Figure 1 shows a flow diagram of the study’s inclusions. During the study period, 264 out of 1,735 patients were diagnosed with FGR. The accuracy of the expected date of birth and gestational weeks was confirmed by referring to the ultrasound images of crown rump length, the last menstrual period, and the date of embryo-transfer if the woman conceived after in vitro fertilization. After excluding 10 patients with chromosomal abnormality, 25 with congenital anomaly, and 48 with multiple gestation, 181 patients with FGR were enrolled. Among these, 88 cases were sFGR: 41 were fullterm and 47 were preterm. The children in these 47 cases were diagnosed with sFGR before birth, and their actual body weights at birth were below the 3^rd^ percentile. Among the 47 cases of preterm sFGR, 15 never had their DQ measured and 6 did not have their DQ assessed during the 1- and 2-year corrected ages. Ultimately, 26 patients were included in the present study as the sFGR group. No patients were diagnosed with TORCH syndrome in the sFGR group. Randomly selected 26 cases of gestational age-matched preterm birth with normal fetal growth were defined as the appropriate for gestational age (AGA) group. The sFGR and AGA groups were selected during the same study period.

### Developmental evaluation

The Kyoto Scale of Psychological Development (KSPD) test was performed by trained testers to assess the children’s neurodevelopment. The KSPD is the most standardized developmental test for Japanese children and covers the following three areas: Postural-Motor (P-M: fine and gross motor functions), Cognitive-Adaptive (C-A: non-verbal reasoning or visuospatial perceptions assessed using materials such as blocks, miniature cars, and marbles), and Language-Social (L-S: interpersonal relationship, socializations, and verbal abilities) ^(9)^. The developmental age for each area is estimated according to the sum score obtained in each of the three sections. An overall developmental age is also obtained. The DQ was calculated by dividing these developmental ages by the child’s chronological age and then multiplying it by 100.

### Statistical analyses

The data are presented as the median (range). Statistical analyses were performed using GraphPad Prism 8 (GraphPad Software, San Diego, CA, USA). Fisher’s exact test was used for categorical variables, whereas the Mann-Whitney *U* test was used for continuous variables. Spearman’s rank correlation was used to evaluate possible associations. A p-value of <0.05 was considered statistically significant.

## Results

### Clinical characteristics

Table 1 shows the clinical backgrounds and pregnancy outcomes of all the cases. There were no significant differences in the maternal age, parity, mode of delivery, and sex of neonates between the two groups. The gestational age at delivery was approximately the same between the AGA and sFGR groups. The fetal body weight and fetal body weight Z score at birth were significantly lower in the sFGR group than in the AGA group. Similarly, fetal height and head circumference at birth and their Z scores were also lower in the sFGR group. No significant difference was noted in the umbilical artery pH and Apgar scores between the groups. In the sFGR group, 19 (73%) patients were diagnosed with hypertension disease of pregnancy, which was higher compared with the 6 (23%) diagnosed patients in the AGA group.

**Table 1.** Patients’ Characteristics and Pregnancy Outcomes.

	AGA (n = 26)	sFGR (n = 26)	*P* value
Age	36 (27-40)	34 (27-42)	0.17
Parity Primipara (n)	12	16	0.4
Multipara (n)	14	10	
Mode of delivery Vaginal (n)	7	2	0.14
C-section (n)	19	22	
Sex of neonate Male (n)	13	14	1
Female (n)	13	12	
Gestational age at delivery	31w4d (25w4d-36w4d)	31w1d (25w2d-36w3d)	1
Fetal body weight (g)	1700 (840-2594)	928 (316-1836)	<0.0001
Z score of body weight	0.078 (−0.94-1.3)	−2.7 (−1.9-−5.0)	<0.0001
Fetal height (cm)	42 (31-49)	35.8 (23.5-43)	0.0003
Z score of height	−0.06 (−1.14-4.03)	−2.22 (−0.28-−4.62)	<0.0001
Fetal head circumference (cm)	29 (24.2-33.5)	25.9 (18.5-31)	0.0005
Z score of head circumference	0.23 (−1.60-3.13)	−1.40 (0.0085-−4.02)	<0.0001
Umbilical artery pH	7.321 (7.016-7.493)	7.259 (7.148-7.432)	0.16
Apgar score at 1 minute	5 (1-9)	6 (1-9)	0.76
Apgar score at 5 minutes	8 (2-9)	8 (3-10)	0.75

AGA: appropriate for gestational age, sFGR: severe fetal growth restriction, C-section: Cesarean-section

In the sFGR group, FGR was recognized at 26 (18-34) weeks of gestation, and once FGR was diagnosed, the diagnostic criteria were met until the time of delivery in all cases. The duration of fetal growth arrest was 21 (0-56) days. Pregnancy was terminated only when there was maternal indication or when one or more of the following findings were observed: repeated deceleration seen in the cardiotocographs, loss of fetal movement, or an absent or reversed a-wave in the fetal ductus venosus Doppler waveform.

With regard to neonatal complications, the following conditions were found. In the AGA group, there were cases of intraventricular hemorrhage (n = 5), periventricular leukomalacia (n = 2, one of them was cerebral palsy), and pervasive developmental disorders (n = 4). In the sFGR group, there were cases of cerebral palsy (n = 2, one of them was intraventricular hemorrhage), pervasive developmental disorders (n = 5), and chronic lung disease (n = 3). There were no cases of necrotizing enterocolitis, sepsis, or blindness in either group.

### DQ scores

**Figure 2.**
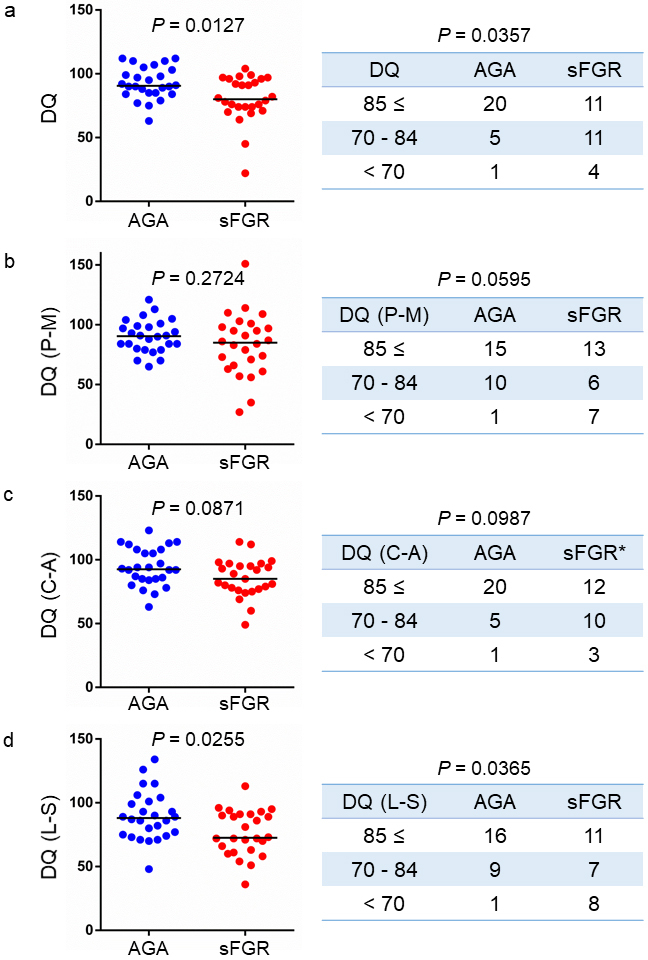
Comparison of the DQ scores of children during the 1- and 2-year corrected ages in the AGA and sFGR groups. DQ: developmental quotient, AGA: appropriate for gestational age, sFGR: severe fetal growth restriction, P-M: Postural-Motor area, C-A: Cognitive-Adaptive area, L-S: Language-Social area (n = 26 in each group, *One data point is missing).

The DQ scores during the 1- and 2-year corrected ages of the children in the AGA and sFGR groups were compared. The overall DQ scores of the children in the sFGR group were significantly lower than those in the AGA group (80 vs. 90.5, *P* = 0.0127) (Figure 2a, left panel). Based on the DQ score, the children were assessed as follows: normal (≥85), borderline (70-84), and delayed (<70). The right panel in Figure 2a shows the number of children in each of these three categories. Moreover, there was a significant difference in the proportions based on these categories between the two groups (*P* = 0.0357).

Subsequently, we compared the DQ scores between the two groups in each of the three areas (P-M, C-A, and L-S). In P-M and C-A, there was no significant difference in the DQ scores between the sFGR and AGA groups (Figure 2b and 2c). However, the DQ scores in the sFGR group were significantly lower than those in the AGA group in L-S (72.5 vs. 88, *P* = 0.0255) (Figure 2d, left panel). The DQ scores in L-S were also significantly different among the three areas (Figure 2d, right panel).

### Clinical factors associated with DQ scores in the sFGR group

**Figure 3.**
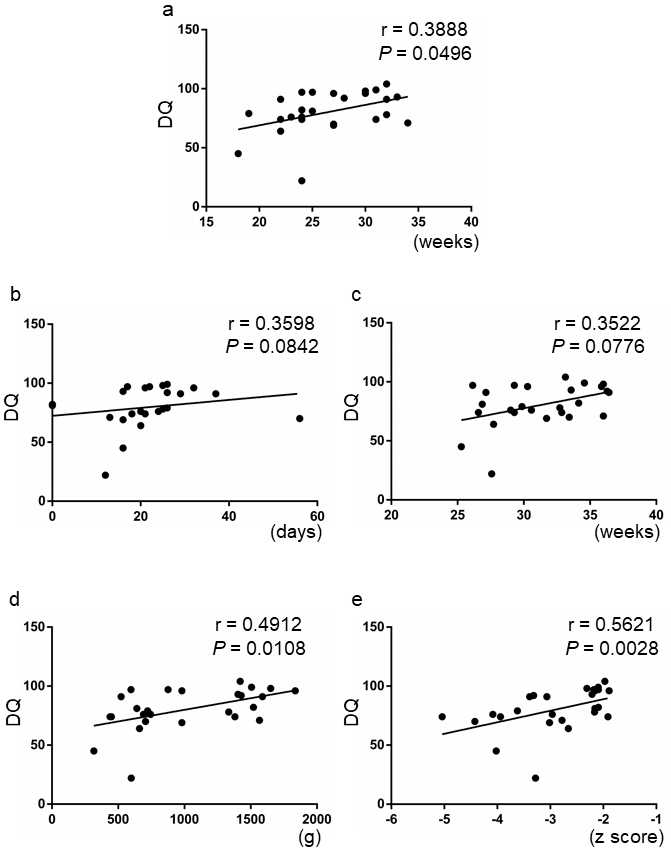
Correlation between the DQ scores and clinical factors in the sFGR group. A: Gestational age at the diagnosis of FGR. B: Duration of fetal growth arrest. C: Gestational age at birth. D: Fetal body weight at birth. E: Z score of fetal body weight at birth. sFGR: severe fetal growth restriction (n = 26).

The correlation between the DQ scores and other clinical factors was also examined. Although the timing of the diagnosis of FGR was slightly correlated with the DQ scores (r = 0.3888, *P* = 0.0496), the duration of fetal growth arrest and gestational age at birth did not correlate with the DQ scores (r = 0.3598, *P* = 0.0842, and r = 0.3522, *P* = 0.0776) (Figure 3a, 3b, and 3c). Meanwhile, both fetal body weight and fetal body weight Z score at birth significantly correlated with the DQ scores (r = 0.4912, *P* = 0.0108, and r = 0.5621, *P* = 0.0028) (Figure 3d and 3e).

## Discussion

The present study analyzed the neurodevelopmental outcomes during the 1- and 2-year corrected ages of preterm-born infants in sFGR and AGA cases. Results showed that children diagnosed with sFGR have poorer overall DQ scores in the assessment using the KSPD. Notably, children in the sFGR group only had significantly lower DQ scores in L-S than those in the AGA group among the three areas measured by the KSPD. Moreover, there was a significant correlation between the DQ scores and fetal body weight or fetal body weight Z score at birth in the sFGR group. There have been studies regarding the neurodevelopmental outcomes of very low birth weight infants using the KSPD in Japanese cohorts. However, these studies present heterogeneous groups of children: preterm infants without FGR, preterm infants with FGR, and fullterm infants with FGR ^(10), (11), (12)^. To the best of our knowledge, this is the first and sole study wherein the neurodevelopmental outcomes of children with preterm sFGR in Japan was clarified using the KSPD.

Infants delivered at less than 37 weeks of gestation due to sFGR have two serious concerns: prematurity due to preterm birth and small physique due to FGR and consequent SGA. In contrast to preterm AGA infants, the complex interplay of these two factors in the case of infants born preterm due to FGR determines the short-term life prognosis and long-term neurodevelopmental outcomes. Specifically, there may be difficulties in characterizing the neurodevelopmental outcome of preterm FGR infants due to the many factors that determine it. Preterm birth itself is known to be associated with neurocognitive impairments ^(13)^; however, it is currently unclear whether prematurity or FGR has a stronger effect on the children’s long-term neurodevelopmental outcome. Our data indicated that preterm birth with FGR is associated with worse DQ scores than mere preterm birth. It may also be implied, despite the small number of cases, that the effect of prematurity on the DQ scores may be stronger up to a certain point of gestational age; the influence of FGR may be more significant afterwards.

Meanwhile, our results are in line with the conclusions drawn from the large systematic review and meta-analysis conducted by Sacchi et al ^(6)^. The study, which included 52,822 children, revealed that cognitive outcomes in childhood were significantly poorer in children with FGR and diagnosed as SGA than in AGA children. Furthermore, the results were similar, regardless of whether the children were born preterm or fullterm. In our case series, there were 41 patients with sFGR born after 37 weeks of gestation, and only 9 of them were followed up long-term for neurodevelopmental outcomes. This may presumably be due to the favorable short-term prognosis for the child’s development. However, there should be long-term monitoring of the neurodevelopment of these children, considering that the neurodevelopment of children with FGR is impaired, even at fullterm ^(6)^.

Even though previous studies have shown unfavorable neurodevelopmental outcomes in children with low birth weight or FGR ^(6), (7)^, its specific features have not been known. Here, the findings of this study revealed the characterized neurodevelopmental outcomes of children born prematurely with sFGR: lower DQ scores in L-S. In the KSPD, development in the L-S area between the ages of 1 and 2 years is assessed as follows: (a) whether children can play ball with the examiner, (b) point at an indicated object such as a dog or car, and (c) answer the names of objects such as flowers and an umbrella. Our results are in partial concordance with the study results of Mukhopadhyay et al. Their results showed that the prevalence of language and visuomotor developmental delay at the corrected age of 2 years was high in infants whose birthweight was less than 1250 g ^(14)^. In addition, SGA infants were at higher risk of this delay ^(14)^. The exact mechanism as to why only the DQ scores in L-S are low in preterm infants with FGR is yet to be clarified. However, considering the findings of this study, care for these infants should focus more on helping them develop in the L-S area. FGR infants, whether born preterm or not, should receive follow-up care and appropriate rehabilitative training after birth.

We employed the KSPD to assess the developmental levels of children, since it is the most widespread and familiar method of testing in Japan, although it has not been standardized in English. Apart from fine and gross motor function, it is essential to have a method that is rooted in the language and culture that the children are exposed to in order to assess language and social development precisely. Furthermore, it has already been shown that the developmental features on the KSPD in the Japanese cohort are well correlated with those on the Bayley III scale, which is a globally acknowledged method for the assessment of developmental outcomes ^(15)^. Therefore, the results obtained in this study have the potential to be extended to non-Japanese children.

The findings of this study have emphasized new implications for the management and optimal timing of delivery in sFGR cases. At present, preterm sFGR cases are managed primarily with the aim of achieving a good short-term perinatal outcome. This means that fetuses are monitored closely using biophysical profile scores, umbilical artery Doppler, ductus venosus Doppler, and cardiotocography and are delivered to preempt serious fetal acidosis or intrauterine fetal death ^(4), (5)^. In practice, however, there are cases in which fetal growth is extremely slow or almost arrested even though fetal well-being is not that bad. In such cases, termination of pregnancy may be an option from the point of view of long-tern neurodevelopmental outcomes, specifically if the Z score of the fetal body weight continuously increases with advancing gestation. Nonetheless, the long-term follow-up of children in the Growth Restriction Intervention Trial showed that there were no clinically significant differences in the neurodevelopmental outcomes between the immediate and delayed delivery groups ^(16)^. Meanwhile, fetal circulatory redistribution has been shown to be associated with neurodevelopmental impairment ^(7)^, which may make obstetricians hesitant to take a deferred delivery approach. It could also be implied that the optimal timing of delivery may not be the only determinant of favorable long-term neurodevelopmental outcomes. A follow-up after birth may be more important to support the development of preterm children with FGR.

A major limitation of this study is its retrospective nature and small sample size. However, as this was a single-center study, the management of FGR and judgment about the timing of delivery were based on specific uniform criteria. Considering this, the potential impact of different management strategies for FGR on neurodevelopmental outcomes is negligible. Another limitation is that the demographic information of both the sFGR and AGA groups (i.e., family socioeconomic status or parent’s educational background) was not known; therefore, they might not be homogenous. This could be potentially relevant to children’s neurodevelopmental outcomes. Future studies employing larger prospective studies could be conducted to further understand the features of the developmental outcomes of preterm children with FGR.

In conclusion, the findings of this study indicate that preterm-born children with sFGR have greater neurodevelopmental impairment than preterm-born children without FGR. A characteristic feature of neurodevelopmental delay is low DQ scores in the L-S area in the KSPD assessment. It is notable that instead of gestational age at birth, the fetal body weight and fetal body weight Z score at birth were significantly correlated with the DQ scores, suggesting that these two factors are important in determining the optimal timing of delivery for sFGR cases. Furthermore, it is imperative that all healthcare providers working with preterm-born infants with FGR are aware of these facts. Moreover, continuous long-term follow-up and appropriate interventions after birth are important for children with sFGR to promote neurodevelopment.

## Article Information

### Conflicts of Interest

None

### Acknowledgement

We would like to thank Editage (www.editage.com) for English language editing.

### Author Contributions

All authors contributed to the conception and design of the study. Takahiro Motoki and Yoshitsugu Chigusa collected and interpreted the data and wrote the first draft of the manuscript. Seiichi Tomotaki carried out a professional analysis of the data from the perspective of a pediatrician. Yosuke Kawamura, Mana Taki, and Ken Yamaguchi made an important contribution to the creation of figures and revision of the manuscript. Masaki Mandai and Haruta Mogami supervised the entire process.

### Approval by Institutional Review Board (IRB)

All procedures were performed in accordance with the ethical standards of the responsible committees on human experimentation (institutional and national) and with the 1964 Declaration of Helsinki and its later amendments. Informed consent was obtained from all the patients included in the study. This study was approved by the Ethics Committee of Kyoto University (R3305).
